# Evaluation of the impact of continuous nursing and cluster nursing on carpal tunnel syndrome release surgery

**DOI:** 10.1186/s13018-024-05246-1

**Published:** 2025-01-07

**Authors:** Yang Chen, Ruihua Li, Yongqiang Zhu, Ran Chen

**Affiliations:** Hand Microsurgery 2 Ward Tianjin Hospital, No.406, South Jiefang Road, HexiDistrict, Tianjin, 300211 China

**Keywords:** Carpal tunnel release surgery, Continuous care, Cluster nursing, Hand function recovery, Quality of life

## Abstract

**Background:**

This study aimed to estimate the influence of continuous and cluster nursing on carpal tunnel syndrome (CTS) release surgery.

**Methods:**

Ninety-six patients with CTS were treated in our hospital from November 2019 to December 2021. These patients were randomly divided into two groups of 48 patients. Both groups underwent open carpal tunnel release surgery. The control group received routine nursing care, while the study group received a combination of continuous and cluster nursing interventions. The Boston carpal tunnel question, the Visual Analogue Scale (VAS), the Numerical Rating Scale (NRS), the Pittsburgh Sleep Quality Index (PSQI), Disabilities of the Arm, Shoulder, and Hand questionnaire (DASH) and the Barthel index were used to compare hand function recovery, hand pain, sleep quality, and quality of life between the two groups before and 3 months after surgery.

**Results:**

Both groups experienced improvements in hand function and pain 3 months after surgery. However, the study group demonstrated lower scores in symptom and dysfunction, as well as lower VAS and NRS scores compared to the control group (*P* < 0.05). Additionally, both groups showed an increase in the Barthel and PSQI scores 3 months after surgery. Notably, the study group exhibited higher Barthel scores and lower PSQI scores than the control group (*P* < 0.05).

**Conclusion:**

The use of continuous and cluster nursing in patients undergoing CTS release surgery proves to be advantageous in alleviating hand pain, facilitating hand function recovery, and effectively enhancing sleep quality and overall quality of life for patients.

## Background

Carpal Tunnel Syndrome (CTS) is an orthopedic condition characterized by anterior wrist pain, hand numbness, and weakness. Epidemiological investigations indicate an increasing prevalence of CTS in China, particularly among middle-aged and older women, as well as individuals with a history of strain injury or occupational disease [1]. Delayed or inadequate treatment can lead to severe complications, including muscle atrophy, significantly impacting patients’ quality of life [2].

Conservative treatments, such as oral medications and local injections, are commonly used for patients with mild CTS and can effectively alleviate symptoms. However, surgical intervention, specifically carpal tunnel release surgery, becomes necessary for patients with moderate to severe CTS [3, 4].

**Fig. 1:**
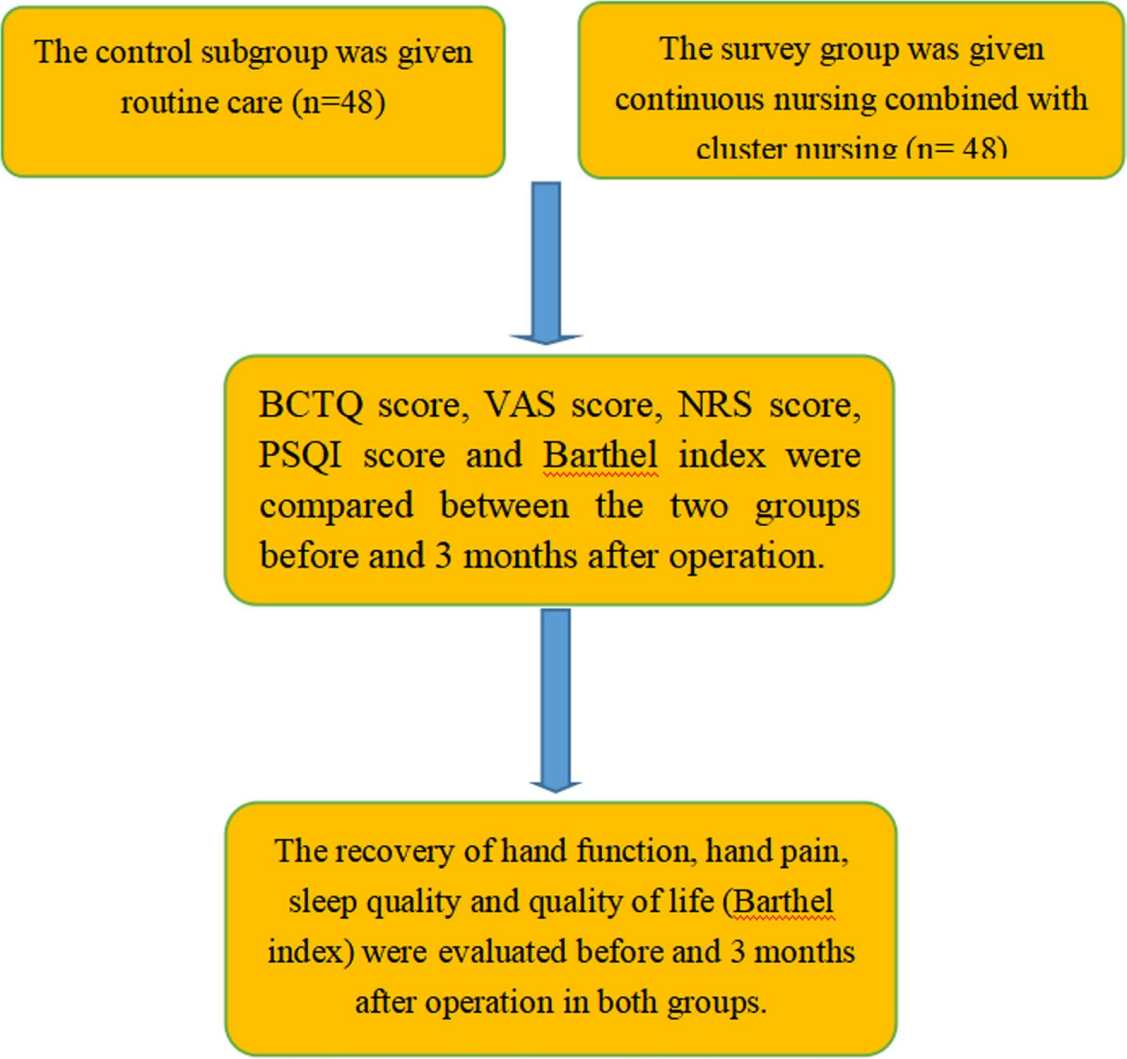
Nursing content

However, many CTS patients experience high levels of tension, anxiety, and other negative emotions due to a lack of awareness of the disease and surgical treatment. These emotional factors can negatively impact treatment adherence and clinical outcomes. Therefore, effective nursing intervention becomes crucial in such cases. Recent reports suggest that cluster nursing during carpal tunnel release surgery can positively impact clinical outcomes [5]. To explore the clinical value of combining cluster nursing with continuous nursing in patients undergoing carpal tunnel release surgery, this study included 96 CTS patients admitted to our hospital from November 2019 and December 2021. The findings of this study are presented Fig. 1.

## Methods

### General information

Ninety-six patients diagnosed with CTS received treatment in our hospital from November 2019 and December 2021. They were randomly divided into two groups of 48 patients. The control group consisted of 22 males and 26 females, aged 43–76 years, with a mean age of 59.51 ± 8.92 years. Twenty patients had left-hand involvement, while 28 had right-hand involvement. The study group comprised 21 males and 27 females, aged 44–75 years, with a mean age of 59.43 ± 8.91 years. Twenty-three patients had left-hand involvement, and 25 had right-hand involvement. No significant differences in the general characteristics were observed between the two groups (*P* > 0.05).

### Inclusion criteria

All patients included in the study met the diagnostic criteria for CTS as outlined in the evidence-based guidelines for CTS management proposed by the American Academy of Orthopedic Physicians [6]. Open carpal tunnel release surgery was performed on all patients who did not have any surgical contraindications. This study received approval from the Medical Ethics Committee of our hospital, and patients’ family members signed informed consent forms.

### Exclusion criteria

Following patients were excluded from the study: Patients with severe coagulation dysfunction, individuals with consciousness disorders or mental illnesses, patients with concomitant hand diseases, individuals with a history of previous wrist surgery, patients who underwent additional physiotherapeutic procedures (neuromobilization, slider exercises, ultrasound, laser biostimulation, cryotherapy) before or after the surgery or nursing intervention, and patients who needed post-operative analgesics.

### Procedure

All patients underwent open carpal tunnel release surgery performed by the same surgical team to minimize intervention bias. The control group received routine nursing care, which included pre-operative patient education, assistance with examinations, and guidance on medication, diet, and rehabilitation exercises as prescribed by the physician. The study group received a combination of cluster nursing and continuous nursing. The cluster nursing subgroup, consisting of attending physicians, head nurses, and nurses, underwent specialized training in cluster nursing. They developed cluster nursing measures based on relevant literature, clinical experience, and the specific condition of CTS patients.

Cluster nursing measures included:

1. Preoperative patient and family education: Patients and their families were provided with clear information about CTS and the surgical plan. They were informed about the surgical procedure, post-operative precautions, and considerations. Psychological interventions were also employed to alleviate anxiety and enhance self-confidence.
2. Postoperative positioning guidance: Patients were instructed to maintain appropriate body positions after surgery.
  - Supine position: Elevate the upper body by about 30°.
  - Standing position: Use a forearm support tool to elevate the affected limb to heart level.
3. Wound care and observation: The affected limb was closely monitored for wound healing. Regular dressing changes were performed. Laser therapy and antibiotic treatment were administered as needed to prevent complications.
4. Limb functional exercise guidance: Patients were instructed to perform simple hand exercises (hand flexion and extension, clenching and loosening) 2–3 times daily for 10–15 min each session.

In addition to cluster nursing, continuous nursing was implemented by establishing nursing files for patients. These files contained basic information and contact details. Three-month follow-up visits were conducted, either in-person or via video call, to assess recovery progress, provide guidance, and schedule future appointments as needed.

### Observations

1. The Boston Carpal Tunnel Questionnaire (BCTQ) was utilized to assess hand function recovery of both groups before and 3 months after surgery. The BCTQ is a 19-item questionnaire that measures two dimensions: symptoms, and dysfunction. Each item is scored on a scale of 1 to 5, with higher scores indicating worse hand function. Studies have shown the BCTQ to be a reliable and valid tool for measuring hand function in patients with carpal tunnel syndrome (Spearman r = 0.71–0.90). Cronbach alpha values ranged from α = 0.80 to 0.90 for the symptoms and from α = 0.88 to 0.93 for the dysfunction [7]. The Chinese version of the BCTQ, used in this study, has been validated for use with Chinese patients and demonstrates excellent reliability (Cronbach’s alpha of 0.92). The results also showed that this version effectively distinguishes between different levels of symptom severity, which is crucial for clinical assessments [8].
2. The Visual Analogue Scale (VAS) and the Numerical Rating Scale (NRS) were employed to evaluate hand pain in both groups before and 3 months after surgery. The total scores for the VAS and the NRS range from 0 to 10, with higher scores indicating more severe pain.
3. Sleep quality was assessed using the Pittsburgh Sleep Quality Index (PSQI). This 24-item self-report questionnaire measures various aspects of sleep. Each item is rated from 0 to 3 with higher scores indicating poorer sleep quality. In the study by Yan et al. (2021), the Chinese version of the PSQI demonstrated good internal consistency with a Cronbach’s alpha of 0.719. Strong correlations were found between PSQI scores and related mental health measures, such as depression, anxiety, stress, and health-related quality of life (HRQoL) [9].
4. Functional status was evaluated using the Barthel Index. This 10-item scale assesses a person’s ability to perform activities of daily living (ADLs). [10]. The total score ranges from 0 to 100 with higher scores indicating greater independence.
5. The scoring method involves summing the points assigned to each item, which reflects the level of assistance required for each activity [3]. Minyu Liang et al. (2024) reported a Cronbach’s alpha of 0.95 for the Barthel Index. This high value indicates excellent internal consistency for the questionnaire used in the study [11].
6. DASH (Disabilities of the Arm, Shoulder, and Hand) questionnaire is a 30-item self-report measure that assesses the impact of upper extremity disorders on daily activities, pain, and overall function. Scores are calculated by summing the scores for each question, resulting in an overall score between 0 and 100 (from less to more disability) [12].
7. DASH score = ((sum of the scores for all completed questions − 1)/(number of completed questions × 4)) × 100
8. The Chinese version of the DASH questionnaire has demonstrated high reliability and validity in studies involving patients with upper extremity musculoskeletal disorders. Key findings indicate strong internal consistency with a Cronbach’s alpha of 0.96 and excellent test–retest reliability, resulting in ICCs close to 0.9. Its validity was confirmed through correlations with other validated measures like the SF-36 and the VAS, demonstrating its ability to capture both physical and social limitations associated with upper extremity disorders [1].

### Statistical analysis

The data were analyzed using SPSS 21.0, and the measurement data were displayed as (‾*x* ± *s*). A t-test was used to determine if the difference between groups was statistically significant (*P* < 0.05).

## Results

### Comparison of hand function levels between the two groups before and 3 months after surgery

**Fig. 2:**
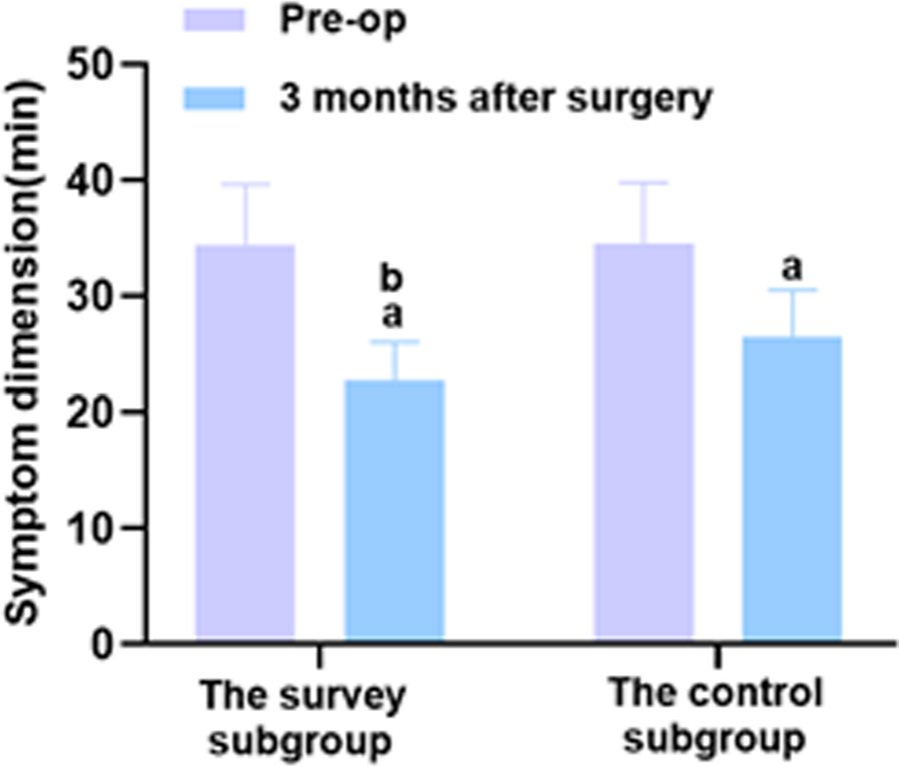
Comparison of symptoms between the two groups before surgery and 3 months after surgery. Versus the same group before surgery, ^a^*P* < 0.05, and versus the control group 3 months after surgery, ^b^*P* < 0.05

**Fig. 3:**
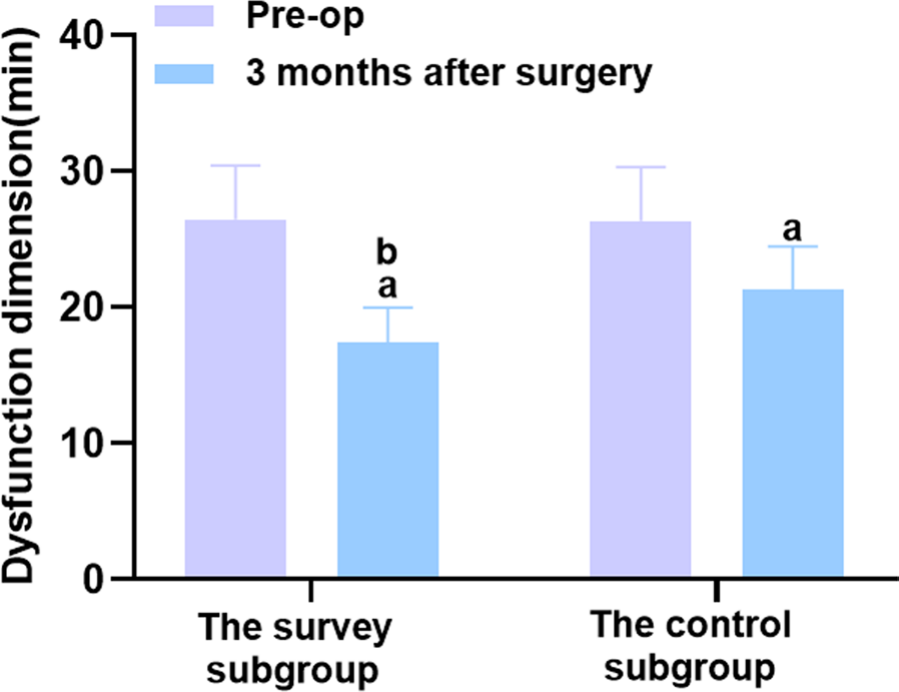
Comparison of dysfunction dimension between the two groups before and 3 months after surgery. Versus the same group before surgery, ^a^*P* < 0.05, and versus the control group 6 months after surgery, ^b^*P* < 0.05. 2.2 Comparison of postoperative sedation effect

The results of the study indicated no significant difference in hand function between the two groups before surgery (P > 0.05). However, both groups experienced improvement in hand function 3 months after the surgery. The study group demonstrated significant differences in the scores of the symptom and dysfunction compared to the control group (*P* < 0.05), as shown by Table 1 and Figs. 2 and 3.

**Table 1:** Comparison of hand function levels between the two groups before and 3 months after surgery (‾*x* ± *s*, min)

Project	n	Symptom dimension		Dysfunction dimension	
		Pre-op	3 months after surgery	Pre-op	3 months after surgery
The study group	48	34.48 ± 5.17	22.67 ± 3.40^a^	26.44 ± 3.96	17.39 ± 2.60^a^
The control group	48	34.62 ± 5.19	26.53 ± 3.97^a^	26.35 ± 3.95	21.28 ± 3.19^a^
*t*		0.132	5.116	0.111	6.548
*P*		0.894	< 0.001	0.911	< 0.001

A marked as the comparison before intervention, *P* < 0.05

### Comparison of hand pain between the two groups before and 3 months after surgery

**Fig. 4:**
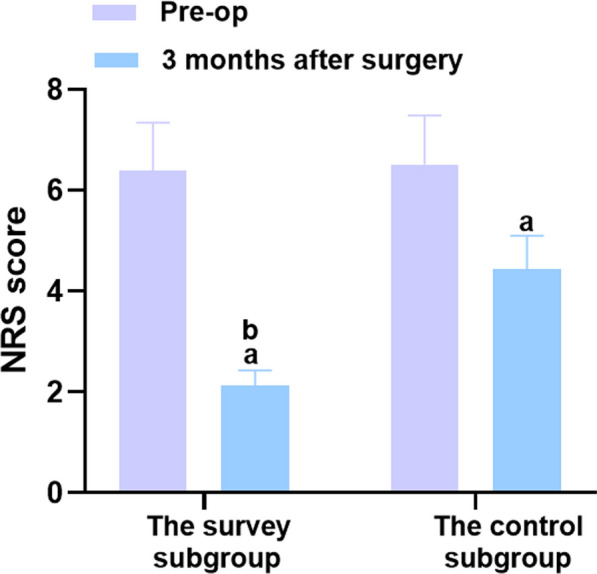
Comparison of the VAS score between the two groups before surgery and 3 months after surgery. Versus the same group before surgery, ^a^*P* < 0.05, and versus the control group 6 months after surgery, ^b^*P* < 0.05

**Fig. 5:**
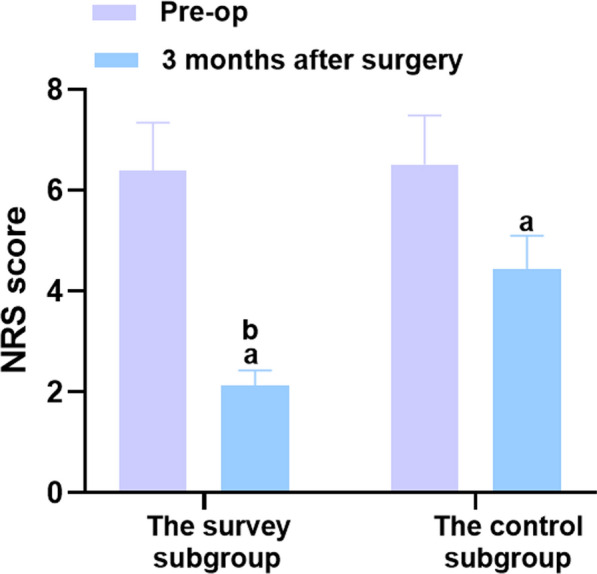
Comparison of the NRS score between the two groups before surgery and 3 months after surgery. Versus the same group before surgery, ^a^*P* < 0.05, and versus the control group 6 months after surgery, ^b^*P* < 0.05

The results indicated no significant difference in the VAS and NRS scores between the two groups before surgery (P > 0.05), but the groups experienced a decrease in the VAS and NRS scores 3 months after surgery, with lower scores observed in the VAS and NRS scores of the study group (*P* < 0.05). Table 2, Figs. 4 and 5 provides more details.

**Table 2:** Comparison of hand pain between the two groups before and 3 months after surgery (‾*x* ± *s*, points)

Project	n	VAS score		NRS score	
		Pre-op	3 months after surgery	Pre-op	3 months after surgery
The study group	48	7.57 ± 1.13	3.65 ± 0.54^a^	6.39 ± 0.95	2.12 ± 0.31^a^
The control group	48	7.43 ± 1.11	5.13 ± 0.76^a^	6.52 ± 0.97	4.44 ± 0.66^a^
*t*		0.612	10.998	0.663	22.043
*P*		0.541	< 0.001	0.508	< 0.001

A marked as the comparison before intervention, *P* < 0.05

### Comparisons of sleep quality and quality of life between the two groups before and 3 months after surgery

**Fig. 6:**
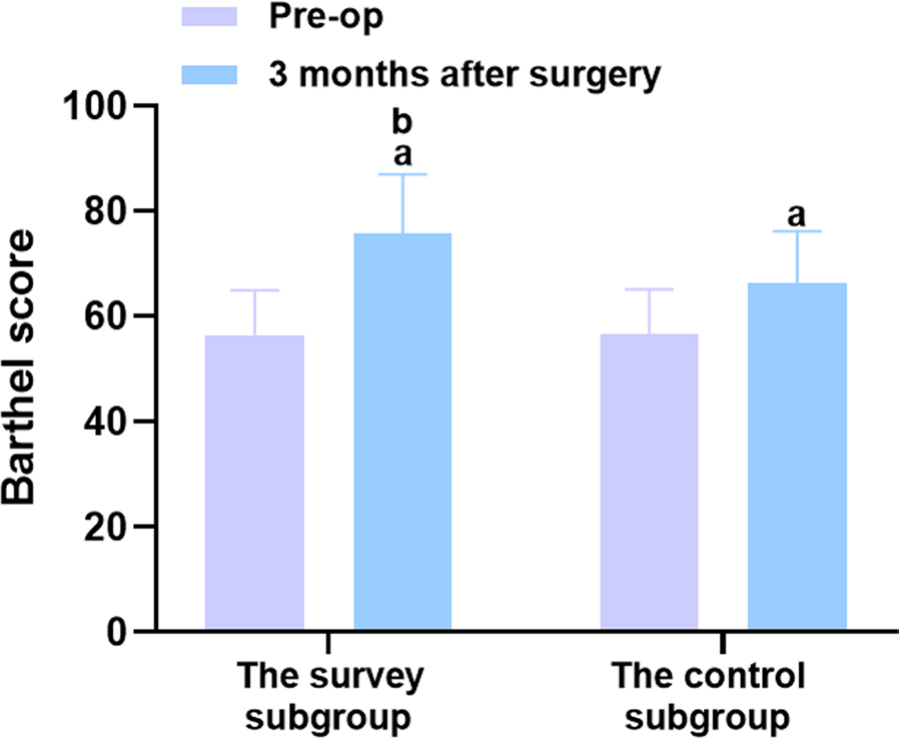
Comparison of the Barthel score between the two groups before surgery and 3 months after surgery. Versus the same group before surgery, ^a^ *P* < 0.05, and versus the control group 6 months after surgery, ^b^ *P* < 0.05

**Fig. 7:**
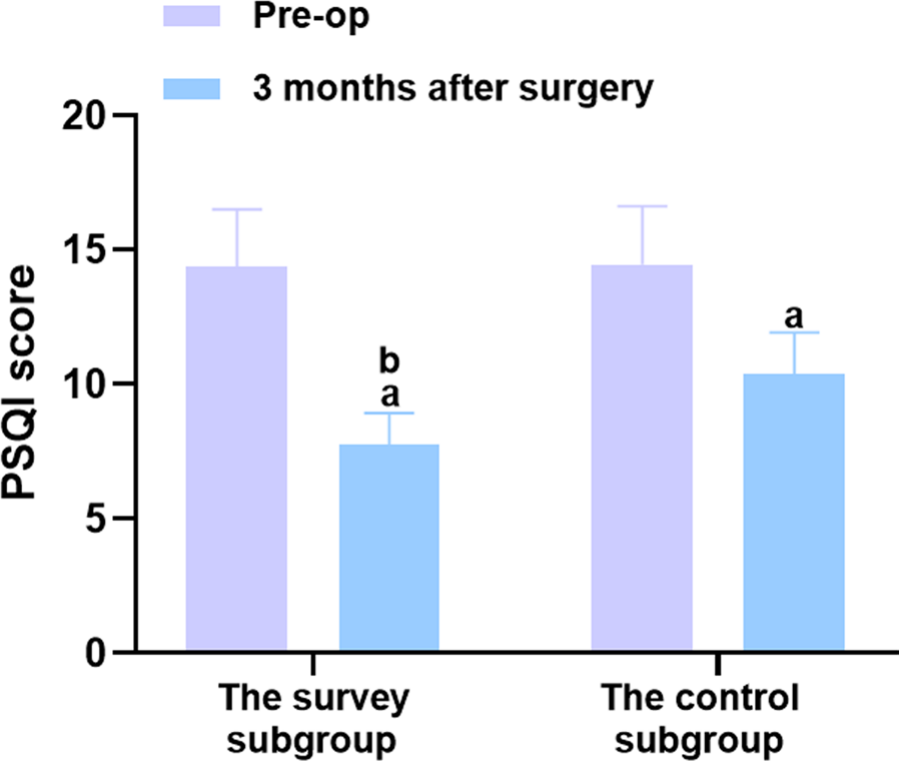
Comparison of the PSQI score between the two groups before surgery and 3 months after surgery. Versus the same group before surgery, ^a^*P* < 0.05, and versus the control group 6 months after surgery, ^b^*P* < 0.05

The results revealed no significant difference in the Barthel and PSQI scores between the two groups before surgery (*P* > 0.05), but the Barthel and PSQI scores in the two groups improved 3 months after surgery. Compared to the control group, the survey subgroup exhibited higher Barthel scores and lower PSQI scores (*P* < 0.05), as shown in Table 3 and Figs. 6 and 7.

**Table 3:** Comparisons of sleep quality and quality of life between the two groups before and 3 months after surgery (‾*x* ± *s*, min)

Project	n	Barthel score		PSQI score	
		Pre-op	3 months after surgery	Pre-op	3 months after surgery
The study group	48	56.48 ± 8.47	75.73 ± 11.35^a^	14.35 ± 2.15	7.78 ± 1.16^a^
The control group	48	56.65 ± 8.49	66.29 ± 9.94^a^	14.46 ± 2.16	10.38 ± 1.55^a^
*t*		0.098	4.334	0.250	9.304
*P*		0.922	< 0.001	0.803	< 0.001

A marked as the comparison before intervention, *P* < 0.05

Table 4 shows the differences in the DASH scores between the survey group and the control group before and 3 months after carpal tunnel release surgery. The survey group, consisting of 48 patients, had a preoperative DASH score of 55.3 ± 12.5, which significantly decreased to 30.1 ± 9.8 3 months after surgery. This improvement of 25.2 ± 3.7 points was statistically significant, with a p-value of less than 0.001, indicating a substantial enhancement in upper limb functionality following the combined continuous and cluster nursing care. Similarly, the control group, also comprising 48 patients, exhibited a reduction in DASH scores from 54.9 ± 11.8 preoperatively to 40.6 ± 10.3 postoperatively. The improvement within the control group was 14.3 ± 2.4 points, which was also statistically significant, with a *p* value of less than 0.001. However, when comparing the postoperative DASH scores between the two groups, the survey group demonstrated a significantly greater improvement than the control group (*p* = 0.002). The preoperative DASH scores between the two groups were not significantly different (*p* = 0.850), confirming that both groups were comparable in terms of baseline disability levels. The significant difference in the postoperative scores between the groups (*p* = 0.002) indicates that the combined nursing intervention in the survey group led to a more considerable improvement in upper limb function and overall recovery compared to the routine nursing care provided to the control group.

**Table 4:** Comparison of the DASH scores between the two groups before and 3 months after surgery (‾*x* ± *s*, min)

Group	Number of Patients (n)	DASH Score (Pre-op)	DASH Score (3 months Post-op)	Improvement	*p*-value
Study Group	48	55.3 ± 12.5	30.1 ± 9.8	25.2 ± 3.7	< 0.001
Control Group	48	54.9 ± 11.8	40.6 ± 10.3	14.3 ± 2.4	< 0.001
Between Groups	96	p = 0.850	p = 0.002		—

Table 5 shows that both the study and control groups experienced similar rates of mild complications after surgery, including infections, numbness, swelling, delayed wound healing, and hematoma. All p-values were greater than 0.05, suggesting no statistically significant differences in the incidence of these mild complications between the two groups. This indicates that mild complications were relatively common among both groups.

**Table 5:** Number of patients who had post-operative complications within a month of surgery

Complication	Study Group (n = 48)	Control Group (n = 48)	p-value
Mild Infection	3 (6.3%)	4 (8.3%)	0.723
Transient Numbness	5 (10.4%)	6 (12.5%)	0.775
Temporary Hand Swelling	7 (14.6%)	8 (16.7%)	0.803
Delayed Wound Healing	2 (4.2%)	3 (6.3%)	0.641
Mild Hematoma	2 (4.16%)	3 (6.3%)	0.985

## Discussion

Carpal Tunnel Syndrome (CTS) is a condition caused by compression of the median nerve in the carpal tunnel, resulting in peripheral nerve entrapment [13]. While mild cases can often be managed conservatively, severe cases may require surgical intervention to alleviate median nerve compression, improve nerve microcirculation, and ultimately reduce clinical symptoms and promote hand function recovery [14–16]. However, surgery can lead to anxiety and stress in patients. As a result, it is essential to implement effective nursing interventions for patients [17, 18].

Cluster nursing, an integrated approach that combines evidence-based nursing practices, can improve the quality of care for patients undergoing carpal tunnel release surgery [19, 20]. The results of this study confirmed that the study group exhibited significantly lower symptoms and dysfunction 3 months after surgery because patients undergoing carpal tunnel release surgery received centralized nursing care, which involved providing them with clear information about their disease and the surgical procedure before the operation. Additionally, addressing psychological factors such as anxiety and tension can enhance treatment adherence and encourage active engagement in rehabilitation exercises, leading to improved hand function levels [21–23].

Furthermore, continuous nursing was implemented in conjunction with cluster nursing. Providing ongoing support and guidance through phone calls and digital platforms could enhance patient adherence to treatment recommendations and facilitated timely intervention [24–26].

Moreover, the study results also demonstrated significant improvements in the VAS, NRS, PSQI scores, and Barthel scores of the study group compared to the control group 3 months after surgery. This indicates that the combination of continuous nursing and cluster nursing effectively alleviated surgical pain, improved sleep quality, and enhanced overall quality of life among patients undergoing carpal tunnel release surgery [27].

These positive outcomes can be attributed to several factors, including:

- Effective pain management: Cluster nursing techniques, such as proper positioning and rehabilitation exercises, can help reduce pain and promote healing [28, 29].
- Enhanced patient education and support: Continuous nursing, including phone calls and digital communication, can provide patients with ongoing support and address their concerns, reducing anxiety and improving adherence to treatment plans.

Consequently, this approach contributed to improvements in sleep quality and overall quality of life [30–32].

## Conclusion

The combination of continuous nursing and cluster nursing for patients undergoing carpal tunnel release surgery proves to be highly beneficial. It effectively alleviates hand pain, promotes hand function recovery, and improves the sleep quality and overall quality of life for patients. Given these positive outcomes, it is highly recommended for clinical promotion and widespread application.

## Limitations

One of the limitations of the present study was the relatively short follow-up period of 3 months. Future studies should consider extending the follow-up period to 6–12 months after surgery. Additionally, the study did not include digital and hand dynamometry to measure nerve and muscle strength. Incorporating these measures in future research could provide more precise and quantitative assessments of functional recovery.

## Data Availability

The datasets used and/or analyzed during the current study were available from the corresponding author on reasonable request.

## Abbreviations

CTS: Carpal tunnel syndrome
BCTQ: Boston carpal tunnel question
VAS: Visual analogue scale
NRS: Numerical Rating Scale
PSQI: Pittsburgh Sleep Quality Index
